# Exploring the Antiviral Potential of Natural Compounds against Influenza: A Combined Computational and Experimental Approach

**DOI:** 10.3390/ijms25094911

**Published:** 2024-04-30

**Authors:** Vladimir Perovic, Kristina Stevanovic, Natalya Bukreyeva, Slobodan Paessler, Junki Maruyama, Sergi López-Serrano, Ayub Darji, Milan Sencanski, Draginja Radosevic, Simone Berardozzi, Bruno Botta, Mattia Mori, Sanja Glisic

**Affiliations:** 1 Laboratory for Bioinformatics and Computational Chemistry, Institute of Nuclear Sciences VINCA, University of Belgrade, 11001 Belgrade, Serbia; kristina.stevanovic@vin.bg.ac.rs (K.S.); sencanski@vinca.rs (M.S.); draga@vinca.rs (D.R.); 2 Department of Pathology, University of Texas Medical Branch, Galveston, TX 77555, USA; 3 Institute for Human Infections and Immunity, University of Texas Medical Branch, Galveston, TX 77550, USA; 4 Infection Biology Laboratory, Department of Medicine and Life Sciences (MELIS), Universitat Pompeu Fabra, Barcelona Biomedical Research Park (PRBB), 08003 Barcelona, Spain; 5 Institut de Recerca en Tecnologies Agroalimentaries (IRTA), Centre de Recerca en Sanitat Animal (CReSA, IRTA-UAB), Campus de la Universitat Autònoma de Barcelona, 08193 Bellaterra, Spain; 6 Department of Chemistry and Technologies of Drugs, Sapienza University of Roma, 00185 Roma, Italy; 7 CLNS—Center for Life Nano Sciences@Sapienza, Istituto Italiano di Tecnologia, 00161 Roma, Italy; 8 Department of Biotechnology, Chemistry and Pharmacy, University of Siena, 53100 Siena, Italy; mattia.mori@unisi.it

**Keywords:** antiviral, natural compounds, influenza, drug resistance, virtual screening

## Abstract

The influenza A virus nonstructural protein 1 (NS1), which is crucial for viral replication and immune evasion, has been identified as a significant drug target with substantial potential to contribute to the fight against influenza. The emergence of drug-resistant influenza A virus strains highlights the urgent need for novel therapeutics. This study proposes a combined theoretical criterion for the virtual screening of molecular libraries to identify candidate NS1 inhibitors. By applying the criterion to the ZINC Natural Product database, followed by ligand-based virtual screening and molecular docking, we proposed the most promising candidate as a potential NS1 inhibitor. Subsequently, the selected natural compound was experimentally evaluated, revealing measurable virus replication inhibition activity in cell culture. This approach offers a promising avenue for developing novel anti-influenza agents targeting the NS1 protein.

## 1. Introduction

Influenza remains a significant global public health issue, causing 5 million severe cases and 290,000 to 650,000 deaths during seasonal outbreaks [1]. Despite the availability of seasonal influenza vaccines, their effectiveness varies, leaving a significant portion of the population vulnerable to the virus [2]. In addition to vaccination, the first line of protection against flu is antiviral medications, such as neuraminidase inhibitors (NAIs) and baloxavir (marboxil), a cap-dependent endonuclease inhibitor of the viral polymerase [3]. Although the present circulating strains have a low rate of NAI resistance, the therapeutic window for NAIs is relatively narrow, and baloxavir (marboxil) drug resistance has been documented [4,5,6]. Favipiravir (T-705) has shown promise against severe novel or reemerging influenza A virus strains when other antivirals are ineffective. However, it is not widely used due to its potential side effects and its limited approval in some countries [7]. Driven by the limitations of current influenza prevention and treatment options, particularly the emergence of novel strains and the threat of drug resistance, researchers are urgently seeking novel and effective anti-influenza therapeutics. Leveraging existing medications’ established safety and efficacy profiles, drug repurposing offers a compelling approach to expedite the development of anti-influenza therapies. Recent reviews have highlighted the potential of this strategy, and investigations into repurposing diverse drug classes [8], among other several antibiotic classes such as macrolides [9] and aminoglycosides [10], have been documented.

The influenza A virus NS1 protein is among the key players in influenza pathogenicity and it stands out for its multi-faceted role in viral replication, immune evasion, and virulence [11,12]. Additionally, it effectively suppresses crucial host antiviral responses, particularly by inhibiting interferon signaling, highlighting its potential as a target for new drug development. Given its well-conserved structure and pivotal role in both replication and pathogenicity, influenza NS1 has garnered significant attention as a promising target for antiviral interventions [11,12,13,14]. Nevertheless, despite the growing interest in NS1, research in this field is still in its early stages, and there are currently no NS1-targeting medicines progressing to preclinical or clinical trials for influenza therapy. Structurally ranging from 230 to 237 amino acids depending on the strain, the NS1 protein forms dimers with two distinct functional domains. The N-terminal domain binds double-stranded RNA, while the C-terminal domain interacts with various cellular proteins, including CPSF30, a key player in processing cellular pre-mRNAs [15]. This interaction disrupts CPSF30-mediated host mRNA processing, contributing to NS1’s immune evasion strategy and allowing the virus to manipulate the host environment to its advantage. Interfering with the interaction between the nonstructural protein NS1 and the cellular protein CPSF30 could potentially serve as a strategy to combat viral infections.

Since ancient times, natural products have served as the foundation for medicines across various cultures. They are often considered safe and effective due to their natural origins and established track record [16]. In terms of toxicity and bioavailability, naturally derived remedies and their ingredients are widely believed to be safer and more effective than substances lacking this natural origin [16]. Due to the diverse range of pharmacophores and stereochemistry found in natural product collections, they could be valuable in identifying possible candidates for challenging screening targets, such as protein–protein interactions [17]. Given that the NS1 protein’s function relies on numerous protein–protein interactions, natural compounds hold substantial promise due to their recognized ability to disrupt such interactions [15,17].

As the in silico equivalent of the high-throughput screening of huge compound databases, virtual screening is an essential aspect of the drug development process, significantly reducing the time and expenses involved in discovering novel medicines [18]. Using this approach, anti-influenza leads have been successfully found from natural chemical databases. In this study, we employed computer-based techniques to investigate the ZINC natural product database, emphasizing the crucial role of natural products in the search for new influenza treatments. The ZINC database [19] is the most extensive, freely available 3D molecular library, with over twenty million commercially accessible chemicals.

This study presents a theoretical framework for the efficient virtual screening of large molecular libraries to identify potential influenza A NS1 protein inhibitors. A promising drug candidate was identified by combining in silico database exploration, using the electron-ion interaction potential/the average quasi valence number (EIIP/AQVN) filter, with ligand-based virtual screening and molecular docking. An experimental validation confirmed the anti-influenza activity of 3-(1H-indol-3-yl)-N-(4-phenylbutan-2-yl)propanamide in cell cultures. Future perspectives include further exploring the capacity of this candidate compound, selected through in silico methods, as an antiviral agent.

## 2. Results

**Figure 1:**
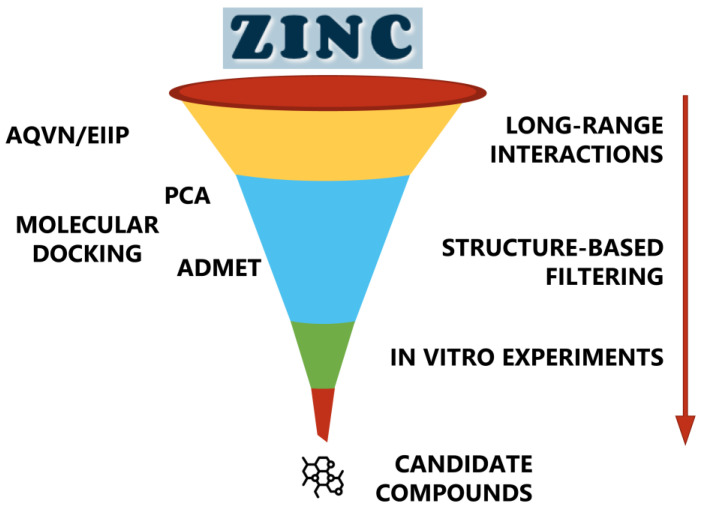
The workflow of the protocol followed in this study.

The virtual screening protocol used in this study involved the application of sequential filters to identify potential influenza A virus NS1 inhibitors. The workflow in Figure 1 details the protocol followed in this study.

**Figure 2:**
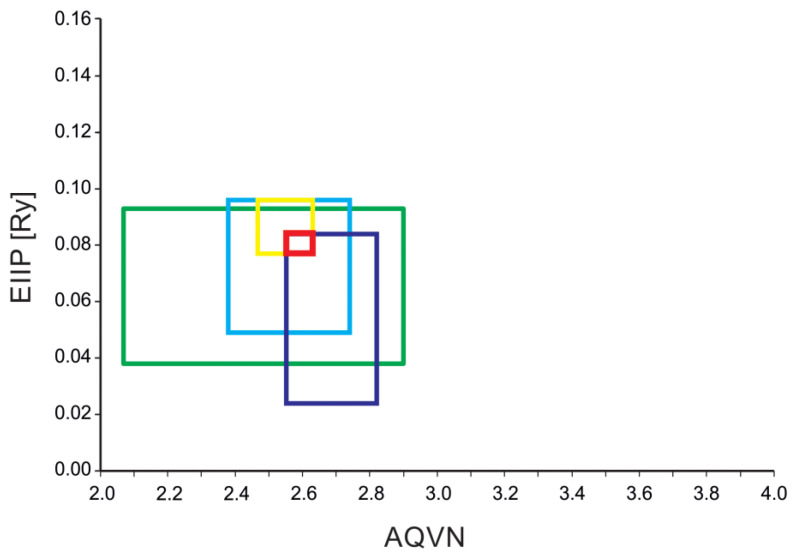
Schematic presentation of the EIIP/AQVN criterion for the selection of candidate anti-influenza NS1 compounds through the virtual screening of molecular libraries (green—NS1 inhibitors, blue—HA inhibitors, purple—aminoglycosides, yellow—macrolides, red—the domain AQVN/EIIP domain employed for virtual screening in this study).

Initially, we conducted virtual screening on the ZINC Natural Product database using the EIIP/AQVN criterion. To commence this screening process, it was necessary to define the screening criteria. Given the absence of an approved NS1 inhibitor, the chosen learning set comprised NS1 inhibitors documented in the existing literature and a compound report card from the ChEMBL (Table S1). More than 80% of the compounds from the learning set (Table S1, Figure 1) were inside the active domain with AQVN and EIIP values within the intervals of (2.069–2.90) and (0.038–0.093), respectively. In light of the extensive range of NS1 inhibitors available, and to refine the domain occupied by NS1 inhibitor drug candidates and enhance the potential for dual targeting against both NS1 and HA by natural compounds, we established the HA inhibitor learning set using sources from the literature within the AQVN/EIIP ranges of (2.380–2.74) and (0.049–0.096), respectively, representing over 80 percent of the analyzed HA inhibitors compounds. Furthermore, based on findings from the literature regarding the potential repurposing of macrolides and aminoglycosides for influenza treatment, we further refined the domain and identified a shared area that could offer multi-target activity. We accomplished this by narrowing down the overlap area within the domain for macrolides with EIIP/AQVN values of 2.467–2.630/0.077–0.096 and for aminoglycosides with values of 2.552–2.820/0.024–0.084. This deliberate refinement of the virtual screening domain increases the likelihood of selecting drug candidates with the potential for multi-target activity. For the final selected shared domain, AQVN/EIIP values of 2.552–2.630/0.077–0.084 were utilized as the criteria for selecting compounds that could serve as NS1 influenza inhibitor candidates. (Figure 2). In this way, a curated collection of 2475 natural compounds was selected for the subsequent in silico steps.

### 2.1. The Principal Component Analysis (PCA) Model

**Figure 3:**
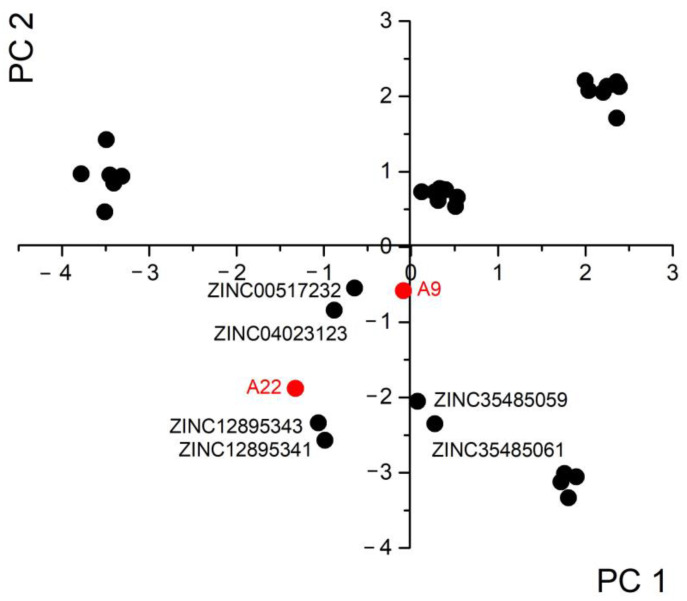
PCA plot of the first two components scores, with marked inhibitors A9 and A22 from the literature [23], and most similar candidate molecules.

The selected candidates from the previous step were filtered according to Lipinski’s rule of five [20]. Subsequently, those that share the same scaffold (two phenyl rings and an amide linking group) as the reported inhibitor molecules were identified [21]. A total of 31 remaining candidate molecules were further classified according to their 3D similarity by calculating the molecular interaction field (MIF) descriptors [22]. The Principal Component Analysis (PCA) method was used to construct the QSAR model based on the acquired data. The first two Principal Components (PC1 and PC2), which encompass most of the model (Table 1), were chosen for the model’s description (Figure 3). The six compounds that resembled inhibitors in the literature the most were selected for structure-based filtering.

### 2.2. Molecular Docking

Selected candidates were then docked into the X-ray crystal structure of the NS1 effector domain from H1N1 influenza A/California/07/2009 (PDBID: 3M5R) (https://www.rcsb.org/structure/3M5R (accessed on 3 March 2024)). The binding site was located at the binding interface between NS1 and the host CPSF30 [24]. To validate the computational model, reference inhibitors (Table S2) were re-docked into the selected receptor structure [25].

**Figure 4:**
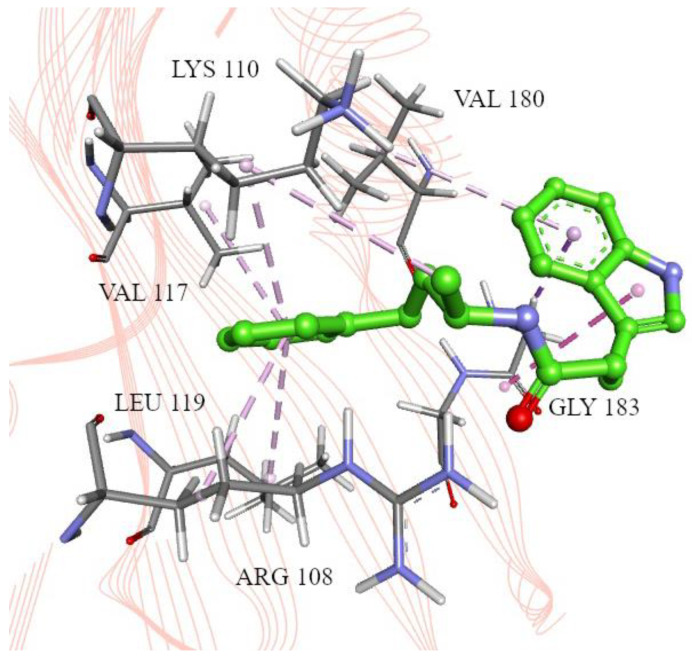
ZINC12895341 (3-(1H-indol-3-yl)-N-[(2S)-4-phenylbutan-2-yl]propanamide) in the H1N1 2009 NS1 effector domain inhibitor binding pocket, with marked interacting amino acid residues. Pink lines: alkyl-π/hydrophobic interactions, purple: π-σ interactions.

**Figure 5:**
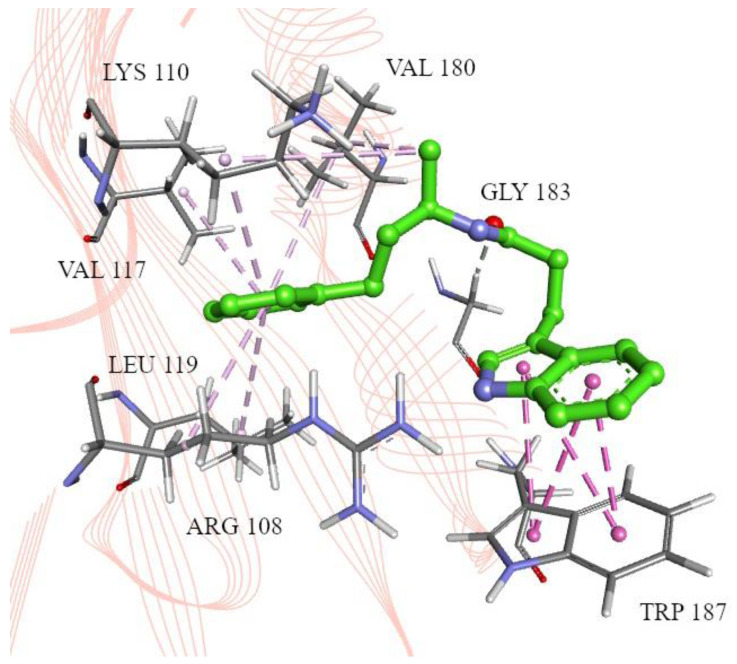
ZINC12895343 (3-(1H-indol-3-yl)-N-[(2R)-4-phenylbutan-2-yl]propanamide) in the H1N1 2009 NS1 effector domain inhibitor binding pocket, with marked interacting amino acid residues. Pink lines: alkyl-π/hydrophobic interactions, magenta: π-π interactions.

Despite the docking results (Table 2) suggesting a potentially weak interaction with the receptor (binding energy of about −6 kcal/mol), a visual inspection revealed that molecules ZINC12895341 (3-(1H-indol-3-yl)-N-[(2S)-4-phenylbutan-2-yl]propanamide) and ZINC12895343 (3-(1H-indol-3-yl)-N-[(2R)-4-phenylbutan-2-yl]propanamide) are positioned (Figure S1) similarly to the inhibitors described in the literature [25]. Owing to the phenyl rings, they can establish favorable interactions in the selected hydrophobic region (Figure 4 and Figure 5). Moreover, the binding free energies’ orders of magnitude also correspond with those of the reference inhibitors [25]. The key amino acids engaged in the interactions are Arg108, Lys110, Val117, Leu119, Val180, and Gly183. The ZINC12895343 enantiomeric form indicates a slightly more favorable binding mode than ZINC12895341 (−6.6 kcal/mol compared to −6.3 kcal/mol, Table 2), most likely thanks to the π-π stacking interaction with the side chain of Trp187. This was validated after rescoring the obtained docking poses for both the candidate compounds and those from the literature. The scoring function was expanded into the individual terms’ contributions to the binding energy (Table S4); the detailed interactions between the compounds and amino acid residues are presented in Table S5 and Figures S2 and S3. The top candidate in both enantiomeric forms has a significantly lower hydrogen bond contribution than the reference compounds, although higher hydrophobic contribution on average. The differences in repulsion and gauss terms between the compound groups are due to the complicated atom–atom network interactions, involving their atom radii and types, where interatomic distances depend on the docking pose. Due to the highly similar binding patterns between the candidate compounds and those in the literature, we state that the docking results support proper selection.

### 2.3. The Absorption, Distribution, Metabolism, Elimination, and Toxicity (ADMET) Prediction

Table S6 displays the estimated ADMET properties of candidate compounds according to the following parameters: molecular weight (MW), number of rotatable bonds (RB), dipole moment (DM), molecular volume (MV), number of hydrogen donors (DHB), number of hydrogen acceptors (AHB), polar surface area (PSA), octanol/water partition coefficient (log P), aqueous solubility (log S), apparent Caco-2 cell permeability (PCaco), number of likely primer metabolic reactions (PM), percentage of human oral absorption (%HOA), violations of rules of three (VRT) and five (VRF), blood–brain barrier permeability parameter (QPlogBB) [26], and Multi-Parameter Optimization score for drugs targeting the Central Nervous System (CNS MPO) [27]. The violations of Lipinski’s [20] and Jorgensen’s [28] rules are shown together with the ADME parameters. Based on the ADMET features, selected compounds may have good pharmacokinetic profiles and may be promising candidates for treating influenza A viruses.

### 2.4. In Vitro Efficacy Testing of 3-(1H-Indol-3-yl)-N-(4-phenylbutan-2-yl)propanamide against H1N1 and H3N2 Influenza A Viruses

**Figure 6:**
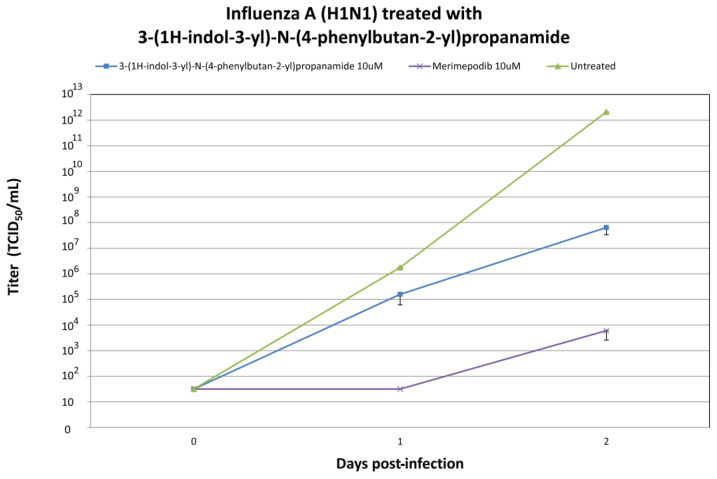
Influenza A/CA/07/2009 (H1N1) viral titers at 0, 1, and 2 days post-infection (dpi) after treatment with the 3-(1H-indol-3-yl)-N-(4-phenylbutan-2-yl)propanamide with the indicated drug concentrations. Ten micromolar (10 μM) merimepodib was used as a positive control. The results are plotted as the means of triplicate observations, with standard deviations shown.

**Figure 7:**
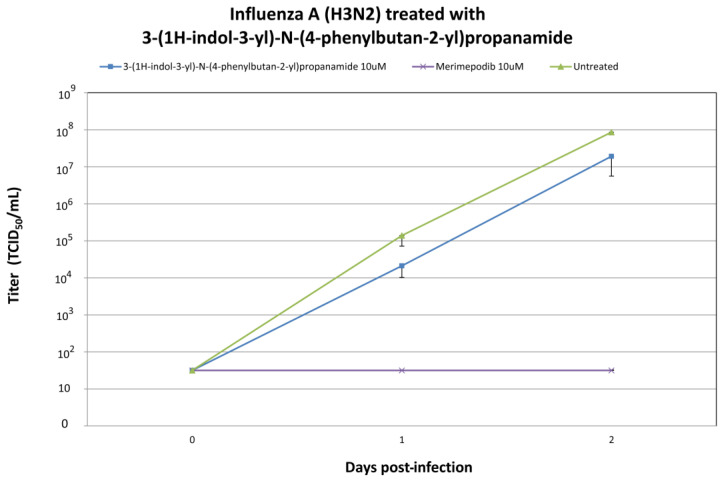
Influenza A/NY/55/04 (H3N2) viral titers at 0, 1, and 2 days post-infection (dpi) after treatment with the 3-(1H-indol-3-yl)-N-(4-phenylbutan-2-yl)propanamide with the indicated drug concentrations. Ten micromolar (10 μM) merimepodib was used as a positive control. The results are plotted as the means of triplicate observations, with standard deviations shown.

We evaluated the in vitro antiviral efficacy of 3-(1H-indol-3-yl)-N-(4-phenylbutan-2-yl)propanamide, the top candidate identified through screening the ZINC database for potential anti-influenza compounds [29]. When 3-(1H-indol-3-yl)-N-(4-phenylbutan-2-yl)propanamide was introduced to cells infected with H1N1 or H3N2 influenza A viruses, it led to a significant reduction in the production of infectious virus. Treatment with 10 μM 3-(1H-indol-3-yl)-N-(4-phenylbutan-2-yl)propanamide resulted in significant reductions in H1N1 viral titers at +1 and +2 days post-infection (Figure 6). A total of 10 μM 3-(1H-indol-3-yl)-N-(4-phenylbutan-2-yl)propanamide treatment resulted in significant reductions in viral titers at +1 and +2 day post-infection. Treatment with 10 μM 3-(1H-indol-3-yl)-N-(4-phenylbutan-2-yl)propanamide resulted in significant reductions in H3N2 viral titers at +1 and +2 day post-infection (Figure 7). As a positive treatment control, influenza A virus was premixed with 10 µM of merimepodib, an IMPDH inhibitor with known antiviral activity against a variety of viruses, including influenza A virus [30,31]. In the experiments with influenza A/CA/07/2009 (H1N1) and A/NY/55/04 (H3N2), it was shown that 3-(1H-indol-3-yl)-N-(4-phenylbutan-2-yl)propanamide inhibits influenza virus production (Figure 6 and Figure 7).

## 3. Discussion

With its ability to circulate among various host populations, the influenza virus poses a persistent global health threat, leading to unpredictable animal and human outbreaks. Due to the increasing use of licensed antiviral medications resulting in the emergence of drug-resistant strains, the current strategies to prevent and treat influenza A and B virus infections are proving inadequate [1]. The influenza A virus NS1, pivotal in viral replication and immune evasion, emerges as a crucial drug target with considerable potential to contribute significantly to the fight against influenza. It achieves this vital role by participating in numerous protein–protein and protein–RNA interactions. Specifically, the NS1 protein physically associates with CPSF 30 kDa within influenza virus-infected cells, preventing CPSF from binding to the RNA substrate, inhibiting 3′ end cleavage and polyadenylation in host pre-mRNAs, and, thus, blocking host transcription [15,32]. Therefore, we focused on disrupting the protein–protein interaction between the nonstructural protein NS1 and the cellular protein CPSF30 with small-molecule inhibitors as a promising approach to thwart viral infections.

Natural product collections are known for their diverse pharmacophores and extensive stereochemistry, making them valuable for identifying potential compounds for challenging screening targets like protein–protein interactions. Due to their proven ability to disrupt such interactions, natural compounds were selected as ideal candidates for inhibiting the CPSF30-NS1 interaction. These compounds offer a promising solution, as they provide potential drug candidates with low toxicity and widespread availability for treating influenza. In our research, we utilized natural products sourced from the ZINC database, underscoring their potential to form the foundation for new agents with improved efficacy and enhanced tolerability in treating infectious diseases.

In the pursuit of innovative strategies to reduce drug resistance and pandemic virus threats, in silico approaches or computer-aided drug design have opened up numerous opportunities for identifying potential novel lead compounds for various diseases, including infectious diseases [18]. As highlighted in ref. [23], numerous predictive computational approaches are being introduced to target influenza viruses. One of the described approaches is the concept of long-range interactions, which we applied in the current study [31]. As previously demonstrated for molecular targets in diverse pathological contexts, molecules sharing similar AQVN and EIIP values tend to interact with common therapeutic targets [31]. Consequently, this observation has given rise to the EIIP/AQVN criteria, which are employed for the virtual screening of molecular libraries in search of compounds exhibiting similar therapeutic properties [33]. Previous investigations have validated the effectiveness of the EIIP/AQVN approach in identifying inhibitors against a range of viral targets, including influenza [34,35]. These findings were further verified experimentally [34,36,37].

In this study, we employed a virtual screening protocol designed to identify potential inhibitors of the influenza NS1 protein. This process entailed the systematic application of sequential filters. We began by implementing an initial filter based on the EIIP/AQVN, proceeding with PCA analysis, and subsequently conducting molecular docking on the comprehensive ZINC Natural Product database. However, we needed to establish precise screening criteria to start this screening endeavor. We curated a learning set by drawing from documented NS1 inhibitors in the existing literature and leveraging the compound report card from ChEMBL (Table S1). Given the available diverse in silico array of NS1 inhibitors, our objective was to narrow down the domain occupied by potential NS1 inhibitor drug candidates. To enhance the likelihood of multi-targeting against both NS1 and different influenza targets using natural compounds, we further constructed the NS1 inhibitor learning set from the literature. Inspired by promising findings in the literature suggesting the repurposing potential of macrolides [9] and aminoglycosides for influenza treatment [10], we linked these to the published AQVN/EIIP domains established for these compounds and undertook further refinement [38]. This endeavor led us to identify a shared domain with the potential for multi-target activity. This deliberate refinement of our virtual screening domain significantly enhances the prospect of selecting drug candidates capable of engaging in multi-target activity against influenza A viruses. Still, these candidates may also offer additional benefits in combating secondary bacterial infections, a common influenza complication, during seasonal and pandemic outbreaks [39].

Furthermore, through the utilization of ligand-based virtual screening, candidates were chosen based on their proximity to the centroid in the PCA model. This model relied on variables derived from MIF descriptors of compounds within the learning set, making their pharmacophore similarity a key criterion for selection. Subsequently, a structure-based approach was employed, enabling the docking of the previously selected compounds into crystal structures as the next step.

Looking at the graph of PCA scores (PC1 against PC2), we observe that compounds are concentrated into several groups. ZINC12895343 and ZINC12895341, 3-(1H-indol-3-yl)-N-(4-phenylbutan-2-yl)propanamide enantiomers, were found to exhibit the highest level of molecular similarity to the most effective known inhibitor, A22 (Figure 2 and Table 2). Certainly, they are clustered around both inhibitors used for constructing the PCA model (Figure 2). A22 disrupts the interaction between NS1 and CPSF30 by binding to the CPSF30-binding pocket in many strains of influenza A virus, indicating its potential as an antiviral agent [25].

Together with the significant molecular similarity found in the QSAR model, molecular docking revealed that the selected candidates also fit best into the hydrophobic pocket identified as essential for NS1 inhibition. Despite the modest differences in positioning between the enantiomeric forms, the method of action and binding energies are very similar. Thus, it was demonstrated that the compound in question potentially attenuates virus replication by disrupting CPSF30–NS 1 interaction. Therefore, it was selected for further experimental testing.

In the in vitro experiments with influenza A/CA/07/2009 (H1N1), it was shown that 3-(1H-indol-3-yl)-N-(4-phenylbutan-2-yl)propanamide inhibits influenza virus production (Figure 5).

While investigating the natural origins of 3-(1H-indol-3-yl)-N-[(1R)-1-methyl-3-phenyl-propyl]propanamide, several resources were consulted. These resources included the NPBS (Natural Products and Biological Sources) database, which links natural products to various plants, bacteria, fungi, and marine organisms [40]. Unfortunately, the NPBS yielded no matches for this specific compound. We further explored the KNApSAcK family database [41], which offers compound information alongside details about the source plant family and species. Again, no corresponding entry for 3-(1H-indol-3-yl)-N-[(1R)-1-methyl-3-phenyl-propyl]propanamide was identified within KNApSAcK. It is important to note that the compound does appear in open databases like ZINC and COCONUT [42], which classify it as a natural product. However, these databases lack information regarding the compound’s specific source.

Our study highlights the promising antiviral activity of 3-(1H-indol-3-yl)-N-[(1R)-1-methyl-3-phenyl-propyl]propanamide, a candidate compound identified via screening the ZINC database. This compound emerged as a candidate due to its structural similarity to A22, a known influenza A virus inhibitor. The in silico part of our study suggested a mechanism similar to A22, indicating 3-(1H-indol-3-yl)-N-[(1R)-1-methyl-3-phenyl-propyl]propanamide’s potential to disrupt the critical NS1-CPSF30 interaction, potentially inhibiting viral replication. In further in vitro experiments, we demonstrated 3-(1H-indol-3-yl)-N-[(1R)-1-methyl-3-phenyl-propyl]propanamide’s efficacy in significantly reducing viral titers for both H1N1 and H3N2 strains. The antiviral activity observed in these experiments encourages the further exploration of the potential of this in silico-selected candidate compound as an antiviral agent.

## 4. Materials and Methods

### 4.1. Data Preparation

A library of natural products that contain 1 × 105 compounds was downloaded from the ZINC database in a processed format [19]. This database can be accessed via the following website: http://zinc15.docking.org (accessed on 31 October 2022). The learning set was created to define the predictive criterion for selecting influenza A virus NS1 inhibitor candidates (Tables S1 and S3). Since there is currently no approved influenza A virus NS1 inhibitor, the selected learning set consisted of NS1 inhibitors reported in the literature and a compound report card from the ChEMBL (Table S1). The learning set for HA inhibitors was derived from the previously published literature (see Table S3 for details). A selected set of 2475 natural compounds was downloaded from the ZINC database [19] in 3D sdf format, and the corresponding SMILES and molecular formulas were isolated. Structures of reported NS1 inhibitors were obtained using BIOVIA Draw 2021 [43] and exported in 3D sdf format. Crystal structures of the NS1 effector domain from A/California/07/2009 (H1N1) were downloaded from the RCSB PDB database (PDBIDs: 3M5R and 3LZG) [44].

### 4.2. EIIP/AQVN Filter

The average quasi valence number (AQVN) and the electron–ion interaction potential (EIIP) [45], generated from the general model pseudopotential [46], determine the specific recognition and targeting between interacting biological molecules at distances greater than 5 Å [33]:

Z* = ∑_i=1,m_(n_i_ Z_i_/N) (1)

EIIP = 0.25 Z* sin(1.04 π Z*)(2)

where Z* stands for AQVN, Z_i_ is the valence number of the i-th atomic component, n_i_ is the number of atoms of the i-th component, m is the number of atomic components in the molecule, and N is the total number of atoms. EIIP values are expressed in Rydberg units (Ry).

AQVN and EIIP account for the unique physical characteristics defining long-range interactions between biological molecules [33]. The biological activity of organic molecules (such as their mutagenicity, carcinogenicity, toxicity, antibiotic, and cytostatic activity) is strongly correlated with their EIIP and AQVN [45].

### 4.3. PCA Model

Both learning set compounds and candidate molecules were imported into Pentacle software (version 1.06 for Linux) [47] in a 3D sdf format. There they were oriented towards principal moments of inertia and protonated there at a physiological pH. GRIND descriptors were calculated using diverse molecular interaction field (MIF) probes [22]. The essential nonbonded interactions were represented by the probes: DRY (hydrophobic interactions), O (hydrogen bond acceptor), N1 (hydrogen bond donor), and TIP (molecular shape descriptor). GRID with step 0.5 was the method for MIF computation, while the discretization algorithm was AMANDA [48] with a scale factor of 0.55. The encoding method was MACC2 with weights set to DRY: −0.5, O: −2.6, N1: −4.2, and TIP: −0.75. The principle components analysis (PCA) model (with the number of PCA components set to five) was built using the collected GRIND descriptors.

### 4.4. Molecular Docking

After downloading PDB crystal structures, receptor preparation implied the removal of all ligands, ions, and water molecules, as well as protonation in accordance with physiological conditions. For this purpose, BIOVIA Discovery Studio 2021 [43] and ADT Tools 1.5.6 were used [29,49]. Ligand molecules (converted to PDB format) were protonated at pH = 7.4 in VEGA ZZ [50] and geometrically optimized in MOPAC 2016 [51] at the PM7 [52] theory level. A grid box of 24 × 24 × 24 Å was placed to encompass every amino acid residue that interacts with human CPSF30 [24]. The grid box (x, y, and z) center was (−24.3, 40.8, −2.5). All docking results were obtained by using Autodock Vina 1.1.2 [53]. The value of exhaustiveness was 50. The scoring function of Autodock Vina calculates the binding energy as a sum on single atom–atom pair contributions, but the basic equation can be expanded into the following terms:

$$∆G=w_{1}*gauss1{+w}_{2}*gauss2+w_{3}*repulsion+w_{4}*hydrophobic\;term+w_{5}*hydrogen\;term$$

where gauss1 and gauss2 represent the contributions of Van der Waals potential and repulsion term steric clashes, and the final two terms are those of hydrophobic interactions and hydrogen bonds. Coefficients w_1_–w_5_ represent weighting parameters.

Figures were made in BIOVIA Discovery Studio 2017 and Origin 9.0 software.

### 4.5. ADMET Prediction

Using QikProp software in normal mode, the ADMET parameters of the candidate compounds and inhibitors were computed [54]. MarvinSketch 22.9 was used to determine CNS MPO values [55].

### 4.6. In Vitro Efficacy Testing against H1N1 and H3N2 Influenza A Viruses

The experiment began by pre-mixing influenza A/CA/07/2009 (H1N1) virus with 3-(1H-indol-3-yl)-N-(4-phenylbutan-2-yl)propanamide, followed by an hour-long incubation at 37 °C. The same process was repeated for the influenza A/New York/55/04 (H3N2) virus. Madin-Darby canine kidney (MDCK) cells in 12-well plates, reaching 85–95% confluency, were washed twice with serum-free media before being infected with the virus/drug mixtures. Each treatment group was tested in triplicate.

As a positive treatment control, the influenza A virus was premixed with 10 µM of merimepodib, an IMPDH inhibitor with known antiviral activity against various viruses, including influenza [30,31].

After an hour of incubation at 37 °C with 5% CO_2_, cells were washed once with serum-free media, and the appropriate concentration of test drug was added to each well. Negative control wells were mock-infected, while virus control wells were infected but left untreated. The cells were then kept at 37 °C with 5% CO_2_, and samples were collected at 0, 24, and 48 h post-infection, then stored at −80 °C until analysis.

In order to measure the reduction in the production of infectious progeny, each sample was diluted at a 1:10 ratio and used to inoculate cells in 96-well plates with approximately 85–95% confluency to determine viral titers using a 50% tissue culture infective dose (TCID50) assay. Growth curves for each virus were constructed based on individual titers collected at the specified time points.

## 5. Conclusions

Natural compounds are significant reservoirs of innovative, highly effective, and specific candidates for treating influenza diseases. A virtual screening was conducted on the ZINC Natural Product database to identify potential inhibitors of NS1 of influenza A viruses. The most promising candidate compound, 3-(1H-indol-3-yl)-N-[(1R)-1-methyl-3-phenyl-propyl]propanamide, was chosen in silico and validated in vitro as a potential novel treatment agent against the influenza A viruses. Further research is required to elucidate the candidate compound’s antiviral effects in vivo and show whether the compound represents a suitable preliminary candidate for alleviating human and animal influenza infections.

## Figures and Tables

**Table 1:** Statistics of PCA model for H1N1 2009 NS1 effector domain inhibitors. SSX—percentage of the X sum of squares; SSXacc—accumulative percentage of the X sum of squares; VarX—percentage of the X variance; VarXacc—accumulative percentage of the X variance.

Component	SSX	SSXacc	VarX	VarXacc
1	27.45	27.45	24.99	24.99
2	22.81	50.26	21.78	46.77
3	11.14	61.40	10.43	57.20
4	8.87	70.28	8.61	65.81
5	4.52	74.80	4.08	69.89

**Table 2:** Selected candidates from the QSAR model with their docking energies. In the last column, Euclidian distance, PA22 from A22 indicates the level of similarity to this most potent inhibitor.

ZINC ID	Vina Docking Energy (kcal/mol)	P_A22_
ZINC12895343	−6.6	0.529736751
ZINC12895341	−6.3	0.768482715
ZINC04023123	−5.6	1.131189207
ZINC35485059	−6.8	1.409582845
ZINC00517232	−5.9	1.49712743
ZINC35485061	−6.5	1.667747682

## Data Availability

The original contributions presented in the study are included in the article and Supplementary Materials, further inquiries can be directed to the corresponding authors.

## Supplementary Materials

The supporting information can be downloaded at: https://www.mdpi.com/article/10.3390/ijms25094911/s1. References [56,57,58,59,60,61,62,63,64,65,66,67,68,69,70,71,72] are cited in the Supplementary Materials.
