# Supplementary material for: Exploring the Antiviral Potential of Natural Compounds against Influenza: A Combined Computational and Experimental Approach
Source: Int J Mol Sci. 2024 Apr 30;25(9):4911. doi: 10.3390/ijms25094911 (PMC11084791; doi:10.3390/ijms25094911)
Supplement: Supplementary file 1 [file ijms-25-04911-s001.zip › Figure and Tables caption.pdf]

Table S1: The learning set comprised NS1 inhibitors documented in the existing literature and a compound report card from the ChEMBL database.

Figure S1: Reference inhibitors [24] (blue carbon atoms: A9; yellow carbon atoms: A22) with candidate molecules (green carbon atoms: ZINC12895341; pink carbon atoms: ZINC12895343) in the H1N1 2009 NS1 effector domain inhibitor binding pocket.

Figure S2: Literature inhibitor A9 in the H1N1 2009 NS1 effector domain inhibitor binding pocket, with marked interacting amino acid residues. Green lines: hydrogen bonds, pink: alkyl-π/hydrophobic interactions, magenta: π-π interactions.

Figure S3. Literature inhibitor A22 in the H1N1 2009 NS1 effector domain inhibitor binding pocket, with marked interacting amino acid residues. Green lines: hydrogen bonds, pink: alkyl-π/hydrophobic interactions, magenta: π-π interactions.

Table S2: Docking energy values of the reference inhibitors [24].

Table S3: The learning set for HA inhibitors derived from previously published titles in the literature.

Table S4: Table of docking scoring functions for candidate and reference compounds.

Table S5: Comparison of intermolecular contacts between the candidates and reference compounds.

Table S6: Calculated absorption, distribution, metabolism, elimination, and toxicity (ADMET) parameters of the compounds.
