# Supplementary material for: Exploring the Antiviral Potential of Natural Compounds against Influenza: A Combined Computational and Experimental Approach
Source: Int J Mol Sci. 2024 Apr 30;25(9):4911. doi: 10.3390/ijms25094911 (PMC11084791; doi:10.3390/ijms25094911)
Supplement: Supplementary file 1 [file ijms-25-04911-s001.zip › Table_S2.pdf]

**Table S2**. Docking energy values of the reference inhibitors.

| Literature inhibitor [24] | Chemical Structure | Literature Vina binding energy (kcal/mol) [24] | Obtained Vina binding energy (kcal/mol) |
|---|---|---|---|
| A9 | 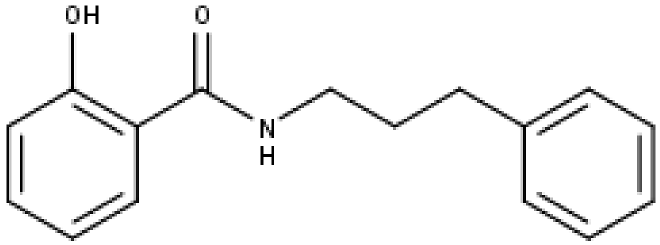 | -6.5 | -6.5 |
| A22 | 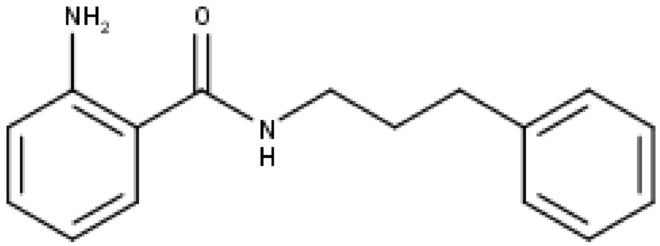 | -6.4 | -6.3 |

References

24. Kleinpeter, A.B.; Jureka, A.S.; Falahat, S.M.; Green, T.J.; Petit, C.M. Structural analyses reveal the mechanism of inhibition of influenza virus NS1 by two antiviral compounds. J Biol Chem. 2018; 293(38):14659-14668. doi: 10.1074/jbc.RA118.004012.

**Biological data:**

A9: Walkiewicz et al. 2011. doi: 10.1099/vir.0.025015-0

A9 and A22: Jablonski et al. 2012. doi: 10.1016/j.bmc.2011.10.026
