# Supplementary figures and images for: Exploring the Antiviral Potential of Natural Compounds against Influenza: A Combined Computational and Experimental Approach
Source: Int J Mol Sci. 2024 Apr 30;25(9):4911. doi: 10.3390/ijms25094911 (PMC11084791; doi:10.3390/ijms25094911)

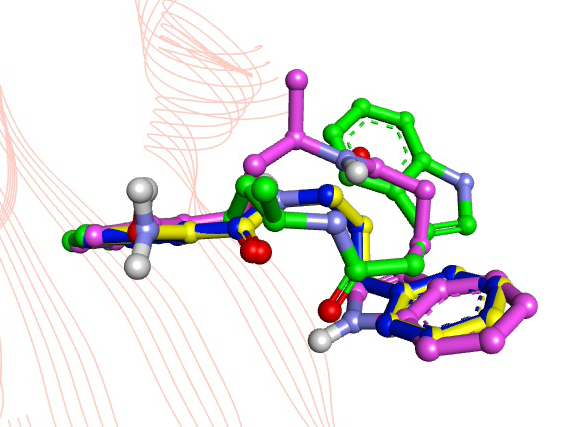

Supplement: Supplementary file 1 [file ijms-25-04911-s001.zip › Figure_S1.png]

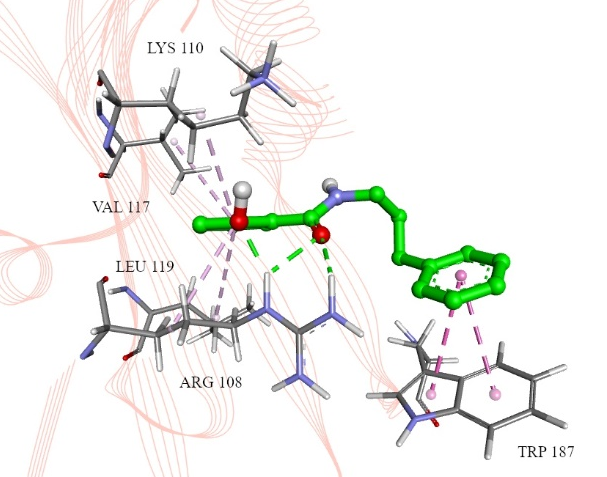

Supplement: Supplementary file 1 [file ijms-25-04911-s001.zip › Figure_S2.png]

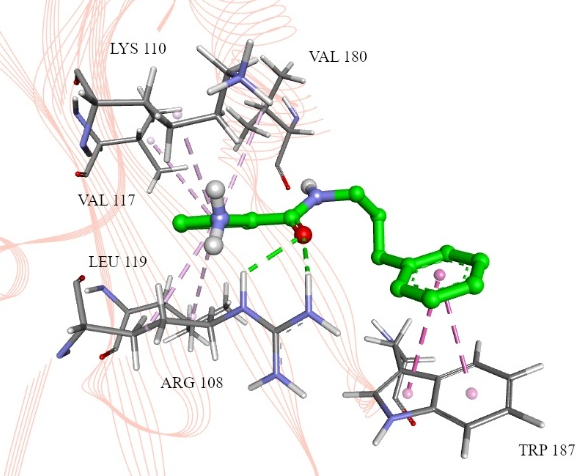

Supplement: Supplementary file 1 [file ijms-25-04911-s001.zip › Figure_S3.png]
